# Molecular Typing of Australian *Scedosporium* Isolates Showing Genetic Variability and Numerous *S. aurantiacum*

**DOI:** 10.3201/eid1402.070920

**Published:** 2008-02

**Authors:** Laurence Delhaes, Azian Harun, Sharon C.A. Chen, Quoc Nguyen, Monica Slavin, Christopher H. Heath, Krystyna Maszewska, Catriona Halliday, Vincent Robert, Tania C. Sorrell, Wieland Meyer, the Australian Scedosporium (AUSCEDO) Study Group

**Affiliations:** * Westmead Hospital, Westmead, New South Wales, Australia; † University of Sydney, Sydney, New South Wales, Australia; ‡ St. Vincent’s Hospital, Sydney, New South Wales, Australia; § Alfred Hospital, Melbourne, Victoria, Australia; ¶ Royal Perth Hospital, Perth, Western Australia, Australia; # University of Western Australia, Perth, Western Australia, Australia; ** Centraalbureau voor Schimmelcultures, Utrecht, the Netherlands; 1 These authors contributed equally to experimental work and data analysis.; 2 Current affiilation: Lille Pasteur Institute, Lille, France

**Keywords:** Scedosporium prolificans, Scedosporium aurantiacum, Scedosporium apiospermum, molecular epidemiology, ITS-sequencing, ITS-RFLP, PCR fingerprinting, AFLP, research

## Abstract

One hundred clinical isolates from a prospective nationwide study of scedosporiosis in Australia (2003–2005) and 46 additional isolates were genotyped by internal transcribed spacer–restriction fragment length polymorphism (ITS-RFLP) analysis, ITS sequencing, and M13 PCR fingerprinting. ITS-RFLP and PCR fingerprinting identified 3 distinct genetic groups. The first group corresponded to *Scedosporium prolificans* (n = 83), and the other 2 comprised isolates previously identified as *S. apiospermum*: one of these corresponded to *S. apiospermum* (n = 33) and the other to the newly described species *S. aurantiacum* (n = 30). Intraspecies variation was highest for *S. apiospermum* (58%), followed by *S. prolificans* (45%) and *S. aurantiacum* (28%) as determined by PCR fingerprinting. ITS sequence variation of 2.2% was observed among *S. apiospermum* isolates. No correlation was found between genotype of strains and their geographic origin, body site from which they were cultured, or colonization versus invasive disease. Twelve *S. prolificans* isolates from 2 suspected case clusters were examined by amplified fragment length polymorphism analysis. No specific clusters were confirmed.

## Abstract 2

Molecular typing showed genetic diversity, dismissed 2 suspected outbreaks of scedosporiosis, and identified multiple strains of the newly described species *S. aurantiacum*.

Despite efforts to identify and eliminate infectious agents, they continue to emerge and reemerge (*1*). Among them, pathogenic fungi contribute substantially to illness and death, especially in immunocompromised patients (*2**,**3*). In contrast to the well-documented opportunists *Candida albicans*, *Cryptococcus neoformans*, and *Aspergillus fumigatus*, the epidemiology and evolution of human infections caused by uncommon but emerging fungi are incompletely understood. Such pathogens include *Scedosporium apiospermum* (teleomorph *Pseudallescheria boydii*) and *S. prolificans,* which are inherently resistant to many antifungal agents (*3**–**5*).

*S. apiospermum* infections occur worldwide, ranging from localized mycetomas to deep-seated disease such as cerebral abscesses (*6**,**7*). This species also colonizes the respiratory tract of ≈10% of patients with cystic fibrosis and chronic suppurative lung disease (*8**–**10*). On the basis of genetic data, a new species, *S. aurantiacum,* was proposed for a subset of isolates previously identified as *S. apiospermum* (*11*). *S. prolificans* infections are geographically more restricted than those caused by *S. apiospermum,* being most prevalent in Australia, Spain, and the United States (*12*–*15*). *S. prolificans* typically causes localized infections in immunocompetent hosts but rapidly fatal disseminated infections in the immunocompromised among whom it has been associated with nosocomial outbreaks (*3*,*12*–*17*).

Since scedosporiosis, in particular that caused by *S. prolificans*, is often refractory to treatment (*3*,*5*), preventive strategies are of paramount importance. However, the epidemiology and mode of transmission of infection are not well understood. Furthermore, the environmental reservoir of *S. prolificans* is unknown. Molecular typing techniques now provide the means to elucidate the epidemiology of *Scedosporium* infections and to investigate potential case clusters (*16*,*18**,**19*). Strains recovered from patients with cystic fibrosis have demonstrated a high degree of genetic variability (*10*,*20*), although a single genetic profile predominated in 1 study (*8*). The degree of genetic variation within *S. prolificans* is more controversial. Two studies have reported low to no intraspecies genetic heterogeneity (*16*,*21*), while a third noted substantial genetic diversity (*19*). The results of these studies may be biased because they included only small numbers of isolates from specific patient populations. Genetic variability among *S. aurantiacum* has not yet been studied.

In this study, we used 4 molecular tools to examine genetic variation among a large number of Australian clinical *Scedosporium* isolates: 1) internal transcribed spacer (ITS)–based restriction fragment length polymorphism (ITS-RFLP) analysis; 2) DNA sequence analysis of the ITS region (selected isolates); 3) PCR fingerprinting using the microsatellite specific core sequence of phage M13; and 4) amplified fragment length polymorphism (AFLP) analysis (isolates from suspected case clusters). We also searched for the newly described species, *S. aurantiacum* and for genetic clustering of strains according to their geographic origin, body site from which they were cultured, and ability to cause invasive disease.

## Materials and Methods

### *Scedosporium* Isolates and Data Collection

A total of 146 *Scedosporium* isolates were studied (Technical Appendix). Forty-six were from the culture collection at the Clinical Mycology Laboratory, Centre for Infectious Diseases and Microbiology Laboratory Services, Westmead Hospital, Sydney, Australia. For these isolates, the following data were captured: demographic information, patient coexisting conditions and risk factors (summarized in the Technical Appendix). The remaining 100 isolates were obtained through a national, prospective, laboratory-based surveillance for scedosporiosis in Australia (the Australian *Scedosporium* [AUSCEDO] Study) from January 2003 to December 2005. The following data were collected: clinical status, risk factor (defined according to published risk factors for scedosporiosis [*4**,**12**–**15*])*,* major comorbidity (based on the International Classification of Diseases, 10th revision, Australian Modification [ICD-10 AM] diagnostic classification system [*22*])*,* isolated species, treatment and outcome. *Scedosporium* strains obtained from a single colony from the primary isolation plate from all patients were forwarded to the Molecular Mycology Research Laboratory, Westmead Hospital, for genotyping. Isolates were identified as *S. prolificans* or *S. apiospermum* by standard phenotypic methods (*23*). Species were confirmed as *S. prolificans* or *S. apiospermum*, and *S. aurantiacum* was identified (*11*) by ITS-RFLP analysis.

### Definitions

An episode of scedosporiosis was defined as the incident isolation of *Scedosporium* spp. from any body site. Two or more episodes, fulfilling the case definition and occurring in different patients that were epidemiologically linked were defined as a potential case cluster. Invasive disease was defined according to the European Organization for Treatment of Cancer/Mycoses Study Group criteria for “definite” or “probable” infection (*24*). All other patients not fulfilling these criteria, including those with “possible” infection were considered colonized. Coincident hospital renovations or construction was considered to be a potential risk factor if major work was undertaken within 3 months before the isolation of *Scedosporium* spp. from a patient.

### Description of 2 Potential Case Clusters

The first potential case cluster involved 8 patients located in the same hematology/hemopoietic stem cell transplant (HSCT) unit at the Alfred Hospital, a large university hospital in Melbourne (September 2000–October 2001; [*15*]). The second consisted of 3 patients located in the same hematology/HSCT ward at Westmead Hospital a major university hospital in Sydney (September 2003–January 2004; unpub. data). Details of the patients involved in these suspected case clusters are summarized in the Technical Appendix. On each occasion, patient isolates were submitted for genetic analyses to inform infection control responses (see Results).

### Genomic DNA Extraction and ITS-RFLP Analysis

Genomic DNA was isolated as described previously (*18*). The ITS1, 5.8S, and ITS2 regions of the rDNA gene cluster were amplified with the primers SR6R and LR1 (Table 1) as described previously (*25*). Amplicons were double digested with the restriction endonucleases *Sau*96I and *Hha*I (New England BioLabs, Ipswich, MA, USA) in accordance with the manufacturer’s recommendations. Digested products were separated by electrophoresis in 3% agarose gels at 100 V for 3–4 h. Banding patterns were analyzed visually.

**Table 1:** Primer and adaptor oligonucleotide sequences used in the study

Primer or adaptor oligonucelotide	Sequence (5′→3′)
rDNA primers	
SR6R	AAGTARAAGTCGTAACAAGG
LR1	GGTTGGTTTCTTTTCCT
M13 fingerprinting primer	
Phage M13	GAGGGTGGCGGTTCT
*Eco*RI adapters	
EA1	CTCGTAGACTGCGTACC
EA2	CATCTGACGCATGGTTAA
*Mse*l adapters	
MA1	GACGATGAGTCCTGAG
MA2	TACTCAGGACTCAT
Preselective primers	
*Eco*RI-T	GACTGCGTACCAATTCT
*Eco*RI-G	GACTGCGTACCAATTCG
*Mse*l-C	GATGAGTCCTGAGTAAC
*Mse*l-G	GATGAGTCCTGAGTAAG
Selective primers	
*Eco*RI-TG	6 FAM-GACTGCGTACCAATTCTG
*Eco*RI-GT	6 FAM-GACTGCGTACCAATTCGT
*Mse*l-CA	GATGAGTCCTGAGTAACA
*Mse*l-GT	GATGAGTCCTGAGTAAGT

### ITS Sequencing

Eleven isolates, representative of each of 3 ITS-RFLP patterns obtained, were selected for ITS sequencing: ITS-RFLP profile A (*S. prolificans*, WM 06.378, WM 06.440, and WM 06.393), ITS-RFLP profile B (*S. apiospermum*, WM 06.389, WM 06.471, and WM 06.497), and ITS-RFLP profile C (*S. aurantiacum,* WM 06.388, WM 06.482, WM 06.495, WM 06.496, and WM 06.498). The ITS region was amplified as described above and commercially sequenced in both directions by using SR6R or LR1 (Table 1) as forward and reverse primers.

### PCR Fingerprinting

The minisatellite-specific core sequence of the wild-type phage M13 was used as a single primer for PCR fingerprinting (Table 1). Amplification reactions were performed as previously described (*18*). Blank control tubes containing all reagents except template DNA were included for each run; each sample was analyzed at least twice. PCR products were separated by electrophoresis on 1.4% agarose gels at 60 V for 14 cm. Strains were defined to be identical if their PCR fingerprinting profiles had a similarity of >97% ( = 1 band difference). Reproducibility of the PCR fingerprinting technique was accessed by re-amplifying 1 strain of each of the 3 *Scedosporium* spp. with all PCR amplifications carried out and re-running those on each gel.

### AFLP Analysis

AFLP analysis was performed as described previously by using either *Eco*RI-GT 6-FAM-labeled and *Mse*I-GT or *Eco*RI-TC 6-FAM-labeled and *Mse*I-CA as selective primer pairs (QIAGEN, Valencia, CA, USA; Table 1) (*26*). All samples were analyzed by using the ABI Prism 3730 system (Applied Biosystems, Foster City, CA, USA). Data collation, fragment sizing, and pattern analyses were performed with GeneMapper software version 3.5 (Applied Biosystems). Only electrophoregram peaks above 1,000 fluorescent units were scored for the presence or absence of bands of the same size (range 50–500 bp) relative to the GeneScan 500 LIZ DNA size standard (Applied Biosystems). Only bands detected in duplicate AFLP experiments were included in the analysis.

### Data Analysis

#### Clinical Data

Statistical analysis was performed by using SPSS version 10.0.07 (SPSS, Chicago, IL, USA) and EpiInfo version 6.0 (Centers for Disease Control and Prevention, Atlanta, GA, USA). Proportions were compared by using the χ^2^ or Fisher exact test. A p value <0.05 was statistically significant.

#### ITS Sequences

ITS sequences obtained from 11 isolates (see above) were aligned with the ITS sequences of the following reference strains obtained from GenBank: *S. apiospermum* CBS 101.22 (accession no. AJ888435), *S. aurantiacum* FMR 8630 (accession no. AJ888440), *S. aurantiacum* IHEM 15458 (accession no. AJ888441) and *S. prolificans* CBS 114.90 (accession no. AY882369) as well as 2 outgroup sequences: *Pseudallescheria africana* CBS 311.72 (accession no. AJ888425), and *Petriella setifera* CBS 164.74 (accession no. AY882352). Phylogenetic analyses were performed by using PAUP* version 4.06.10 (*27*).

#### PCR Fingerprinting Patterns and AFLP Fragments

PCR fingerprinting patterns were analyzed by using the 1D gel analysis module (BioGalaxy [BioAware, Hannut, Belgium]) in BioloMICS version 7.5.30 (BioAware). Images were normalized for lane to-lane differences in mobility by the alignment of patterns obtained on multiple loadings of the 1kb DNA size marker (GIBCO-BRL, Gaithersburg, MD, USA). The unweighted-pair group method by using arithmetic averages and the procedures of Nei and Li (*28*), both implemented in BioloMICS, were used to generate dendograms based on the coefficient of similarity (*29*) between the isolates. In addition, principal coordinate analysis (PcoA; BioloMICS) was conducted to give an overall representation of the observed strain variation. AFLP fragments were analyzed with BioloMICS.

## Results

A total of 146 *Scedosporium* isolates from 120 episodes (119 patients) were studied (Technical Appendix). Demographic data were available for 108 (90%) episodes and coexisting conditions and risk factor data for 115 (95.8%). Most episodes were reported from New South Wales (64.2%), followed by Victoria (19.2%) and Western Australia (9.2%). The male: female ratio was 1.3: 1. The major patient coexisting conditions and known risk factors for scedosporiosis are summarized in the Technical Appendix. Thirty-nine patients (32.7%) had no underlying medical condition. Coincident building construction was noted in 27 cases (22.5%). *Scedosporium* isolates were associated with invasive disease in 46 (38.3%) instances; the remaining 74 (61.7%) were isolated from patients who were colonized (Table 2).

**Table 2:** Selected characteristics for 120 isolations (episodes) of *Scedosporium* spp.

Characteristic*	No*.* (%) *Scedosporium prolificans*, n = 75†	No. (%) *S. apiospermum*, n = 25†	No. (%) *S. aurantiacum*, n = 23†
Male sex	40 (53.3)	12 (48)	8 (34.8)
Risk factor			
Surgery <30 d	3 (4)	1 (4)	–
Trauma	5 (6.7)	–	1 (4.4)
Clinical status‡			
Invasive disease	39 (52)	4 (16)	4 (17.4)
Colonization	36 (48)	21 (84)	19 (82.6)
Body site of isolation			
Blood	18 (24)	–	–
Eye	2 (2.7)	–	1 (4.4)
Skin/soft tissue	4 (5.3)	1 (4)	2 (8.7)
Lung/respiratory tract	15 (20)	10 (40)	12 (52.2)
Ear	4 (5.3)	10	5 (21.2)

*Some patients had *Scedosporium* isolated from more than 1 body site. Dash indicates no data. †Refers to no. episodes in which each species was isolated. The total no. of isolates was 146, comprising 83 *S. prolificans*, 33 *S. apiospermum,* and 30 *S. aurantiacum*. ‡More than 1 *Scedosporium* spp. was isolated from 4 patients.

### Molecular Typing of *Scedosporium* Isolates

All 146 isolates were examined by ITS-RFLP analysis and PCR fingerprinting. ITS sequencing was performed on 11 strains as described above. AFLP analysis was performed only for selected *S. prolificans* isolates, including the isolates of the suspected case clusters and isolates representative of the *S. prolificans* branches identified by PCR fingerprinting (Appendix Figure 1).

#### ITS-RFLP Analysis

**Figure 1:**
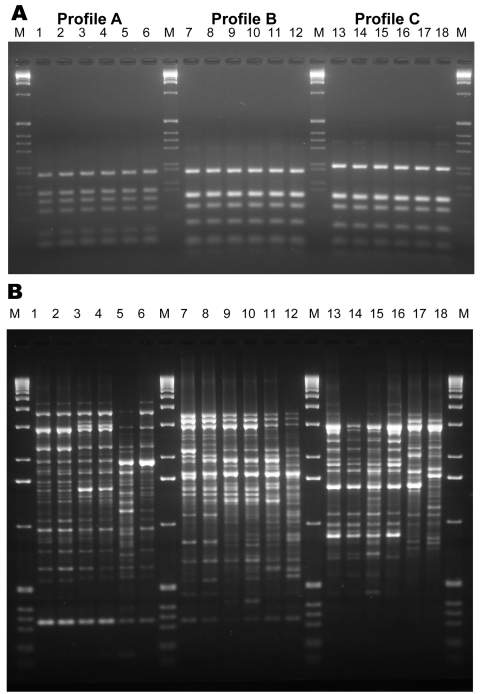
Internal transcribed spacer–restriction fragment length polymorphism (ITS-RFLP) patterns obtained by double digestion with the enzymes *Sau*96I and *Hha*I (A) and of the PCR fingerprinting profiles obtained with the microsatellite specific primer M13 (B) for *Scedosporium prolificans*: lane 1, WM 06.457; lane 2, WM 06.458; lane 3, WM 06.503; lane 4, WM 06.502; lane 5, WM 06.399; lane 6, WM 06.434. *S. aurantiacum*: lane 7, WM 06.495; lane 8, WM 06.496; lane 9, WM 06.386; lane 10, WM 06.385; lane 11, WM 06.482; lane 12, WM 06.390. *S. apiospermum*: lane 13, WM 06.475; lane 14, WM 06.474; lane 15, WM 06.472; lane 16, WM 06.471; lane 17, WM 06.424; lane 18, WM 06.443; lane M, 1-kb marker (GIBCO-BRL, Gaithersburg, MD, USA).

RFLP analysis found 1 RFLP profile specific for *S. prolificans* isolates (ITS-RFLP profile A) and 2 profiles (ITS-RFLP profiles B and C) for isolates previously phenotypically identified as *S. apiospermum* (Figure 1, panel **A**). ITS-RFLP profile B corresponded to *S. apiospermum* and ITS-RFLP profile C to the newly described species, *S. aurantiacum*.

#### ITS Sequencing

Sequencing of the ITS 1, 5.8S, and ITS2 regions of the 11 strains, representative of each of the 3 ITS-RFLP profiles found the following results: BLAST searches against the corresponding GenBank reference sequences identified strains: WM 06.389 (accession no. EF639870), WM 06.497 (accession no. EF639872), and WM 06.471 (accession no. EF639871) (ITS-RFLP profile B) as *S. apiospermum* (96%–99% sequence similarity to strain CBS 101.22). Strains WM 06.388 (accession no. EF639865), WM 06.482 (accession no. EF639866), WM 06.495 (accession no. EF639867), WM 06.496 (accession no. EF639868), and WM 06.498 (accession no. EF639869) (ITS-RFLP profile C) were identified as *S. aurantiacum* (100% sequence identity with strains FMR 8630 and IHEM 15458). Isolates WM 06.393 (accession no. EF639863), WM 06.440 (accession no. EF639864) and WM 06.378 (accession no. EF639862) (ITS-RFLP profile A) were identified as *S. prolificans* (100% identity with strain CBS 114.90).

**Figure 2:**
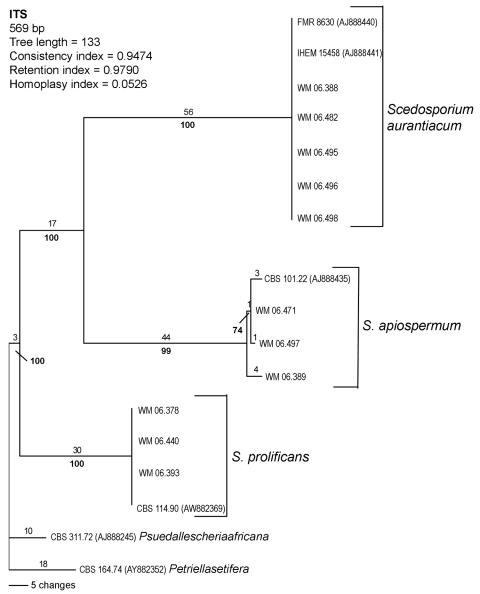
Rooted phylogram (outgroup *Pseudallescheria africana* CBS 311.72 and *Petriella setifera* CBS 164.74), showing the relationships among 11 selected strains representing each obtained internal transcribed spacer (ITS)–restriction fragment length polymorphism pattern and 4 reference strain sequences obtained from GenBank by using PAUP* version 4.06.10 (*29*).

Phylogenetic analysis of the sequences demonstrated 3 distinct clades, the first corresponding to *S. prolificans* as the basal clade. The other 2 corresponded to the 2 more closely related but clearly distinct clades, *S. apiospermum,* and *S. aurantiacum* (Figure 2). *S. apiospermum* showed intraspecies sequence variation of 2.2% compared to *S. aurantiacum* and *S. prolificans,* which displayed no variation.

### Final Identification of *Scedosporium* spp. and Clinical Associations

*S. prolificans* accounted for 75 patient episodes (83 of 146 isolates; 56.9%), *S*. *apiospermum* for 25 (33 isolates; 22.6%), and *S. aurantiacum* for 23 (30 isolates; 20.6%) (Technical Appendix). More than 1 *Scedosporium* spp. was isolated from the same patient in 3 instances: Patient 83: *S. apiospermum* (WM 06.471, WM 06.472, WM 06.474, and WM 06.475) and *S. prolificans* (WM 06.473); patient 91: *S. apiospermum* (WM 06.486) and *S. prolificans* (WM 06.485); and patient 102: *S. apiospermum* (WM 06.500) and *S. prolificans* (WM 06.501) (Technical Appendix). In 6 episodes, the same species was recovered from more than 1 body site in the same patient at the same time (patients 57 [blood, bronchial washing, skin], 73 [blood, sputum], 80 [sputum, bone, wound fluid], 83 [bronchial washing, bronchoalveolar lavage], 118 [pleural fluid, bone, wound fluid, chest tissue], and 119 [blood, skin]; Technical Appendix).

Approximately half (40%–52.2%) of *S. apiospermum* and *S. aurantiacum* isolates were from the respiratory tract/lung compared to 20% for *S. prolificans*. Conversely, all isolates from blood, 57.2% isolates from skin/soft tissue and 66.7% from eye were *S. prolificans* (Table 2). Invasive disease was more likely to be caused by *S. prolificans* than non-*prolificans Scedosporium* spp. (83% versus 17% of isolations; odds ratio (OR) 5.3, 95% confidence interval (CI) 2.0, 14.2, p = 0.002) (Table 2). This association was significant when compared with *S. apiospermum* as well as with *S. aurantiacum* (p<0.05; data not shown). The relative proportions of invasive disease among *S. apiospermum* and *S. aurantiacum* were similar (Table 2). Coincident building construction (27 cases, 22.5%) was more likely to be associated with isolation of *S. prolificans* compared with non-*prolificans Scedosporium* spp. (OR 11.5, 95% CI 2.4, 74.5; p<0.001; data not shown).

### Molecular Epidemiology

#### Strain Typing

**Figure 3:**
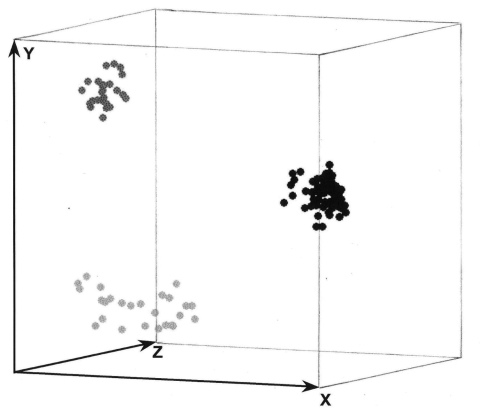
Three-dimensional presentation of the principal coordinate analysis of the PCR fingerprinting data showing 3 distinct clusters which correspond to *Scedosporium prolificans* (black dots), *S. aurantiacum* (dark gray dots), and *S. apiospermum* (light gray dots), with *S. apiospermum* showing the highest genetic variation.

PCR fingerprinting delineated 3 major clusters concordant with *S. apiospermum*, *S. aurantiacum,* and *S. prolificans* (Appendix Figure 1; Figure 1, panel** B**; Figure 3). Clusters corresponding to *S. aurantiacum* and *S. prolificans* were substantially more densely grouped than the *S. apiospermum* cluster (Figure 3).

PCR fingerprinting profiles showed polymorphisms within each of the 3 species, allowing for a clear differentiation, by using a “cut-off point” of >97% similarity. Multiple isolates from the same patient obtained from different anatomic sites (Technical Appendix) had identical or >97% similarity between their PCR fingerprints, except for 1 patient (patient 118). In 8 instances, PCR fingerprinting showed that patients were infected with 2 different strains: (patients 1, 10, 27, 57, 83 99, 118 (Appendix Figure 1, Technical Appendix). For all species, genetic profiles were independent of geographic origin, body site of isolation or whether the patient was infected or colonized (Appendix Figure 1). Profiles were also independent of patient comorbidityity and risk factors for scedosporiosis (data not shown). Intraspecies PCR fingerprinting variation was highest for *S. apiospermum* (58%) followed by *S. prolificans* (45%) and *S. aurantiacum* (28%) (Appendix Figure 1).

#### Examination of Isolates from Suspected Case Clusters

**Figure 4:**
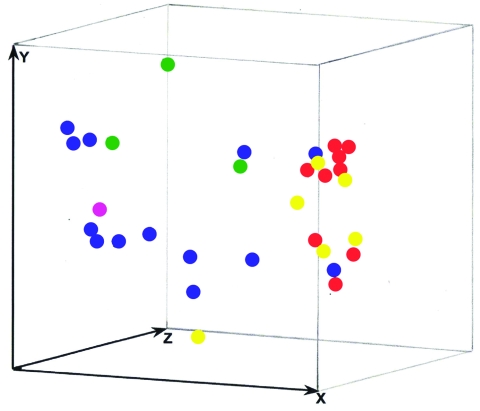
Three-dimensional presentation of the principal coordinate analysis of the combined M13 PCR fingerprinting, amplified fragment length polymorphism (AFLP) primers *Eco*RI-GT and *Mse*I-GT and AFLP primers *Eco*RI-TG and *Mse*I-CA data from the suspected Sydney and Melbourne case cluster isolates and 23 other Australian isolates. None of the investigated isolates showed any epidemiologic connection except 3 isolates obtained from the same patient (nos. 1, 73, 119). Blue dots, Melbourne outbreak isolates; pink dot, Melbourne-related isolate; red dots, Sydney outbreak isolates; green dots, Sydney-related isolates; yellow dots, unrelated Australian isolates.

Twelve isolates from 2 presumptive case clusters of *S. prolificans* infection (Alfred Hospital, Melbourne patients: isolates WM 06.392, WM 06.393, WM 06.395, WM 06.399, WM 06.400, WM 06.401, WM 06.402, and WM 06.405; Westmead Hospital, Sydney patients: isolates WM 06.432, WM 06.434, WM06.457, and WM 06.458; Technical Appendix, as well as 23 additional isolates, representative of the *S. prolificans* branches identified by PCR fingerprinting (Appendix Figure 1) were further investigated by AFLP typing. *S. prolificans* was not isolated from the environment in either setting despite extensive sampling. The AFLP bands were found to be 50–493 bp by using the primers *Eco*RI-GT and *Mse*I-GT (data not shown), and from 52–468 bp by using the primers *Eco*RI-TG and *Mse*I-CA (Appendix Figure 2). These 35 isolates exhibited 32 different AFLP profiles, with isolates from the same patient (patients 1, 73, and 119) showing identical profiles (Appendix Figure 2), confirming the PCR fingerprinting results (Appendix Figure 1). PcoA of the combined AFLP and PCR fingerprinting data demonstrated no clustering of these isolates (Figure 4), which ruled out the possibility of nosocomial transmission.

## Discussion

We examined genetic variation among a large number of population-derived *Scedosporium* isolates across the Australian continent. In line with previously reported genetic variability in the *S. apiospermum/P. boydii* species complex (*30*–*32*), we observed 2 distinct ITS-RFLP patterns among *S. apiospermum* isolates, showing the presence of the newly described species *S. aurantiacum* (*11*). Notably, we have identified by ITS sequencing that *S. aurantiacum* comprised 45% of the current collection of Australian “*S. apiospermum”* isolates and documents genetic variability within *S. aurantiacum*.

Epidemiologic investigation of *Scedosporium* infection requires accurate identification and typing. *S. apiospermum*, *S. aurantiacum,* and *S. prolificans* were clearly distinguished from each other by PCR fingerprinting and ITS-RFLP analysis. This is consistent with previous rDNA sequence-based studies (*30*,*33*,*34*). The observation of 2 distinct genetic groups, corresponding to *S. aurantiacum* and *S. apiospermum,* supports the proposal that *S. aurantiacum* be designated a separate species (*11*). This proposal is also supported by the 5%–10% ITS sequence variation found between *S. aurantiacum* and *S. apiospermum* compared to an absence of intraspecies variation in *S. aurantiacum* and *S. prolificans* and a 2.2% variation in *S. apiospermum* ( *30,32;* current study).

Using PCR fingerprinting, intraspecies variation was greatest (58%) among *S. apiospermum* isolates (Figure 3). This diversity is generally consistent with the high degree of polymorphism (15–20 genotypes) previously found (*10**,**20**,**32*). In contrast, genetic variation was lowest (28%) among the *S. aurantiacum* isolates (Appendix Figure 1; Figure 3). Nevertheless, PCR fingerprinting polymorphisms clearly differentiated all 30 strains (Appendix Figure 1). Further genotyping studies of a greater number of and more geographically diverse *S. aurantiacum* isolates are warranted.

The intraspecies PCR fingerprint variation in *S. prolificans* (45%) was greater than that in *S. aurantiacum* but less than that in *S. apiospermum.* Given that *S. aurantiacum* is phylogenetically more closely related to *S. apiospermum* than to *S. prolificans* ( *11,33;* current study), this result was unexpected. It may be due to different evolutionary pressures acting on the 3 different species or the relatively small numbers of *S. aurantiacum* isolates studied to date. The moderate genetic diversity among *S. prolificans* confirms previous findings (*19*). Despite the observed polymorphisms, PcoA of PCR fingerprint profiles showed dense clustering for *S. prolificans* (Figure 3), which is consistent with the low to absent intraspecies variability in *S. prolificans* found by others (*20*,*21*,*33*). These apparently contradictory findings emphasize the importance of choosing the optimum molecular typing tool with the most appropriate discriminatory power for the organism or species being studied.

The high degree of intraspecies variation detected by PCR fingerprinting and AFLP analysis supports the use of these methods to establish genetic relatedness between isolates recovered from different patients or multiple isolates from the same patient. In comparison, the variation detected by ITS-RFLP analysis and ITS sequencing corresponded to interspecies variation, which makes those techniques ideal for identification of any given isolate to the species level. Individual patients are most likely infected or colonized with genetically distinct strains ( *19–21;* this study). Identical PCR fingerprint or AFLP profiles were noted in multiple isolates recovered simultaneously from different anatomic sites in the same patient (*21*; current study). However, 8 patients were infected or colonized by at least 2 strains as reflected by their different genetic profiles (Technical Appendix). Possible explanations include concomitant infection by multiple strains from which only a restricted number were recovered, or colonization by 1 strain followed by infection or colonization with a second strain of a different genotype. Longitudinal genotyping studies are required to determine the likelihood that persistence of >1 genotypes later leads to clinically important infection or whether the disease is more likely to be caused by an unrelated genotype. In this context, the development of a multilocus sequence typing scheme for *Scedosporium,* as has been developed for *Candida* spp. (*35*), would be of great advantage to overcome interlaboratory reproducibility problems, which are known to be associated with PCR fingerprinting or AFLP data. However, developing such a scheme remains cumbersome due to the current lack of genomic data of *Scedosporium* spp.

For all 3 *Scedosporium* spp., there was no clustering of strains according to their geographic or body site of origin or by their ability to cause invasive disease, which is in agreement with previous findings for *S. apiospermum* (*20*,*30*) and *S. prolificans* (*16*,*17*,*21*). Of note, no specific genotypes were associated with underlying medical conditions or risk factors. Compared with *S. apiospermum* and *S. aurantiacum* *S. prolificans* was more frequently associated with coincident hospital renovation, and invasive disease, had a greater predilection to cause disseminated infection and was the predominant species isolated from blood and other sterile sites (*12**–**16**,**36*; current study). Our preliminary observations indicate that the epidemiology and clinical relevance of recovering *S. aurantiacum* may be similar to that of *S. apiospermum. S. aurantiacum* has been reported to colonize the respiratory tract of at-risk patients (*8*).

In addition to PCR fingerprinting, we applied AFLP analysis to investigate the possibility of 2 case clusters caused by *S. prolificans*. AFLP analysis was chosen as an independent technique using 2 combinations of selective primers (Table 1), which have been previously shown to have good discriminatory power for fungal strain differentiation (*26*). Both techniques, previously used to identify outbreak strain clusters in the recent cryptococcosis outbreak on Vancouver Island (*37*), generated in the current situation distinct patterns from all *S. prolificans* isolates except serial isolates obtained from the same patient (Appendix Figure 1, Appendix Figure 2). These findings exclude the occurrence of nosocomial outbreaks or any close relationship with the nonoutbreak isolates, a result similar to those obtained previously (*38*). Overall nosocomial acquisition of infection has been demonstrated in only 2 instances (*16**,**17*). *Scedosporium* spp. have rarely been isolated from hospital air or from indoor or outdoor surface samples (*13*,*39**,**40*, current study), which raises questions about the mode of acquisition by patients and the mechanisms of the selection of this specific fungus as an infectious agent from among the high biodiversity of environmental molds.

In conclusion, ITS-RFLP analysis is a powerful tool for distinguishing between isolates of the new species *S. aurantiacum* and *S. apiospermum.* PCR fingerprinting and AFLP analysis are useful techniques for determining genetic relatedness between *Scedosporium* isolates and for investigating potential case clusters.

## Supplementary Material

Technical Appendix Strains used in molecular typing of Australian Scedosporium isolates*

Appendix Figure 1 Dendogram generated from the PCR fingerprinting profiles obtained with the microsatellite primer M13 for all investigated Scedosporium isolates. The dendogram was designed by using the unweighted pair group method with arithmetic mean and the procedure of Nei and Li (32) in the program BioloMICS version 7.5.30. Pt, patient.

Appendix Figure 2 Dendogram generated by unweighted pair group method with arithmetic mean and the procedure of Nei and Li (32) for the amplified fragment length polymorphism (AFLP) profile obtained from the 2 suspected case clusters and selected 23 other Australian Spedosporium prolificans strains using the primer pairs EcoRI-TG and MseI-CA. None of the investigated isolates showed any epidemiologic connection except the isolates obtained from the same patient (nos. 1, 73, 119). Pt, patient.
