# Erratum: Vol. 14, No. 2

**DOI:** 10.3201/eid1404.074696

**Published:** 2008-04

In the article “Molecular Typing of Australian *Scedosporium* Isolates Showing Genetic Variability and Numerous *S. aurantiacum*” by L. Delhaes et al., Figure 1, p. 286, contained errors. Profile C should have referred to lanes 7–12, and Profile B should have referred to lanes 13–18. The correct version of the figure is available from www.cdc.gov/eid/content/14/2/282-G1.htm.

We regret any confusion this error may have caused.

