# Supplementary material for: Molecular Typing of Australian Scedosporium Isolates Showing Genetic Variability and Numerous S. aurantiacum
Source: Emerg Infect Dis. 2008 Feb;14(2):282–90. doi: 10.3201/eid1402.070920 (PMC2600218; doi:10.3201/eid1402.070920)
Supplement: Technical Appendix — Strains used in molecular typing of Australian Scedosporium isolates* [file 07-0920_Techapp-s1.pdf]

Technical Appendix

Table. Strains used in molecular typing of Australian *Scedosporium* isolates*

Patient	WM no.	Age/Sex	Specimen type	Comorbidity/risk factor	Status	Building renovation	City/State	Date of isolation†	Species	Comments	Strain profile
1	WM 06.373	2/M	Sputum	Cystic fibrosis	C	No	Townsville, QLD	1999	*S. prolificans*		Strain 1a
	WM 06.374	2/M	Tracheal aspirate	Cystic fibrosis	C	No	Townsville, QLD	1999	*S. prolificans*		Strain 1b
	WM 06.375	2/M	BAL	Cystic fibrosis	C	No	Townsville, QLD	1999	*S. prolificans*		Strain 1a
	WM 06.372	4/M	BAL	Cystic fibrosis	C	No	Adelaide, SA	01/04/2001	*S. prolificans*		Strain 1a
2	WM 06.376	51/M	Tissue (chest)	Trauma	I	No	Canberra, ACT	2000	*S. prolificans*		
3	WM 06.377	45/F	Bronchial wash fluid	Solid organ malignancy	C	No	Brisbane, QLD	2000	*S. prolificans*		
4	WM 06.378	61/M	Tissue (cornea)	Trauma	I	Yes	Melbourne, VIC	2000	*S. prolificans*		
5	WM 06.379	77/M	Tissue (leg)	Trauma	I	Yes	Sydney, NSW	2000	*S. prolificans*		
6	WM 06.380	20/M	Synovial fluid	HSCT	I	Yes	Melbourne, VIC	09/2000	*S. prolificans*	Suspected Melbourne outbreak	
7	WM 06.381	27/F	Blood	Hematological malignancy	I	Yes	Perth, WA	06/1994	*S. prolificans*		
8	WM 06.383	43/M	CSF	Hematological malignancy	I	No	Perth, WA	07/1991	*S. prolificans*		
9	WM 06.384	28/M	Blood	Trauma	I	No	Perth, WA	08/1989	*S. prolificans*		
10	WM 06.385	29/M	Ear swab	None	C	No	Sydney, NSW	05/11/2001	*S. aurantiacum*		Strain 10a
	WM 06.386	29/M	Ear swab	None	C	No	Sydney, NSW	06/11/2001	*S. aurantiacum*		Strain 10b
11	WM 06.387	73/M	Nasal mucosa	Chronic sinusitis	C	No	Sydney, NSW	01/12/2001	*S. aurantiacum*		
12	WM 06.388	NA†	Tissue (corneal)	Trauma	I	No	Perth, WA	09/2001	*S. aurantiacum*		
13	WM 06.389	50/M	Sputum	None	C	No	Wauchope, NSW	09/2001	*S. apiospermum*		
14	WM 06.390	64/M	Skin	Diabetes mellitus	C	No	Perth, WA	03/2001	*S. aurantiacum*		
15	WM 06.391	42/F	Skin/nail	None	C	No	Sydney, NSW	09/2001	*S. prolificans*		
16	WM 06.392	46/F	Sputum	Cystic fibrosis	C	Yes	Melbourne, VIC	10/2001	*S. prolificans*	Suspected Melbourne outbreak	
17	WM 06.393	23/M	Synovial fluid	HSCT	I	Yes	Melbourne, VIC	09/2000	*S. prolificans*	Suspected Melbourne outbreak	
18	WM 06.394	38/F	Nasal fluid	None	C	Yes	Melbourne, VIC	01/2002	*S. prolificans*		
19	WM 06.395	40/M	Tissue (nasal mucosa)	Hematological malignancy	C	Yes	Melbourne, VIC	10/2001	*S. prolificans*	Suspected Melbourne outbreak	
20	WM 06.396	25/M	Blood	Hematological malignancy	I	Yes	Melbourne, VIC	05/2002	*S. prolificans*		

Patient	WM no.	Age/Sex	Specimen type	Comorbidity/risk factor	Status	Building renovation	City/State	Date of isolation†	Species	Comments	Strain profile
21	WM 06.397	85/M	Sputum	Hematological malignancy	C	Yes	Melbourne, VIC	05/2002	*S. prolificans*		
22	WM 06.398	44/F	Blood	Hematological malignancy	I	Yes	Melbourne, VIC	05/2002	*S. prolificans*		
23	WM 06.399	28/F	Blood	Hematological malignancy	I	Yes	Melbourne, VIC	11/2001	*S. prolificans*	Suspected Melbourne outbreak	
24	WM 06.400	33/M	Blood	HSCT	I	Yes	Melbourne, VIC	10/2001	*S. prolificans*	Suspected Melbourne outbreak	
25	WM 06.401	57/F	Intervertebral disk	HSCT	I	Yes	Melbourne, VIC	10/2001	*S. prolificans*	Suspected Melbourne outbreak	
26	WM 06.402	49/F	Synovial fluid	Trauma	I	Yes	Melbourne, VIC	09/2000	*S. prolificans*	Suspected Melbourne outbreak	
27	WM 06.403	66/F	Skin	None	C	No	Sydney, NSW	09/2001	*S. prolificans*		Strain 27a
	WM 06.404	66/F	Skin	None	C	No	Sydney, NSW	09/2001	*S. prolificans*		Strain 27b
28	WM 06.405	47/M	BAL	Lung transplantation	I	Yes	Melbourne, VIC	10/2001	*S. prolificans*	Suspected Melbourne outbreak	
29	WM 06.406	67/F	Blood	Hematological malignancy	I	No	Perth, WA	03/1992	*S. prolificans*		
30	WM 06.407	43/M	Cerebrospinal fluid	Hematological malignancy	I	No	Perth, WA	07/1991	*S. prolificans*		
31	WM 06.408	67/F	Blood	Hematological malignancy	I	No	Perth, WA	03/1992	*S. prolificans*		
32	WM 06.409	54/F	Blood	Hematological malignancy	I	NA	Perth, WA	09/2001	*S. prolificans*		
33	WM 06.410	NA	Ear swab	None	C	No	Brisbane, QLD	04/2001	*S. prolificans*		
34	WM 06.412	NA	Bronchial wash fluid	None	C	No	Sydney, NSW	04/2001	*S. prolificans*		
35	WM 06.413	NA	Bronchial wash fluid washings	NA	C	No	Brisbane, QLD	04/2001	*S. prolificans*		
36	WM 06.414	41/M	Bronchial wash fluid	None	C	No	Adelaide, SA	1999	*S. prolificans*		
37	WM 06.415	NA	Tissue (nasal mucoa)	None	C	No	Brisbane, QLD	04/2001	*S. prolificans*		
38	WM 06.416	58/M	Blood	Other immuno-compromise	I	No	Melbourne, VIC	2000	*S. prolificans*		
39	WM 06.417	NA	Tissue (knee)	Recent surgery	C	No	Brisbane, QLD	2000	*S. prolificans*		
40	WM 06.418	NA	Sputum	None	C	No	Sydney, NSW	04/2001	*S. prolificans*		
41	WM 06.420	56/F	Skin	None	I	No	Perth, WA	08/1991	*S. prolificans*		
42	WM 06.424	32/M	Ear swab	None	C	No	Sydney, NSW	14/04/2003	*S. apiospermum*		
43	WM 06.425	20/F	Sputum	Cystic fibrosis	C	No	Sydney, NSW	04/03/2003	*S. aurantiacum*		
44	WM 06.426	63/M	Bronchial wash fluid	Lung transplantation	I	No	Sydney, NSW	10/06/2003	*S. prolificans*		

Patient	WM no.	Age/Sex	Specimen type	Comorbidity/risk factor	Status	Building renovation	City/State	Date of isolation†	Species	Comments	Strain profile
45	WM 06.427	28/M	Sputum	Chronic lung disease	C	No	Sydney, NSW	10/06/2003	*S. aurantiacum*		
46	WM 06.428	59/F	Tissue (sphenoid sinus)	Chronic sinusitis	C	Yes	Sydney, NSW	23/06/2003	*S. apiospermum*		
47	WM 06.429	NA	Skin	None	C	No	Sydney, NSW	21/07/2003	*S. apiospermum*		
48	WM 06.430	66/M	Skin	None	I	No	Sydney, NSW	04/06/2003	*S. apiospermum*		
49	WM 06.431	19/F	Skin	None	C	No	Sydney, NSW	21/07/2003	*S. apiospermum*		
50	WM 06.432	32/M	Blood	Hematological malignancy	I	Yes	Sydney, NSW	22/09/2003	*S. prolificans*	Suspected Sydney outbreak	
51	WM 06.433	56/M	Sputum	Hematological malignancy	I	No	Sydney, NSW	22/09/2003	*S. prolificans*		
52	WM 06.434	67/M	Blood	Hematological malignancy	I	Yes	Sydney, NSW	28/11/2003	*S. prolificans*	Suspected Sydney outbreak	
53	WM 06.435	74/M	BAL	Recent surgery	C	Yes	Sydney, NSW	26/11/2003	*S. apiospermum*		
54	WM 06.436	57/M	Blood	Hematological malignancy	I	Yes	Wollongong, NSW	01/10/2003	*S. prolificans*		
55	WM 06.437	44/F	BAL	Cystic fibrosis	C	No	Sydney, NSW	15/12/2003	*S. apiospermum*		
56	WM 06.438	58/F	Sputum	Lung transplantation	C	No	Sydney, NSW	01/12/2003	*S. prolificans*		
57	WM 06.439	64/M	Blood	Hematological malignancy	I	No	Sydney, NSW	26/12/2003	*S. prolificans*		Strain 57a
	WM 06.440	64/M	Bronchial wash fluid	Hematological malignancy	I	No	Sydney, NSW	26/12/2003	*S. prolificans*		Strain 57b
	WM 06.441	64/M	Skin	Hematological malignancy	I	No	Sydney, NSW	26/12/2003	*S. prolificans*		Strain 57a
58	WM 06.442	58/M	Sputum	Hematological malignancy	I	Yes	Sydney, NSW	02/01/2004	*S. prolificans*		
59	WM 06.443	61/F	Sputum	None	C	No	Sydney, NSW	10/05/2003	*S. apiospermum*		
60	WM 06.444	80/M	Sputum	None	C	No	Sydney, NSW	28/03/2003	*S. aurantiacum*		
61	WM 06.445	62/M	Sputum	Hematological malignancy	I	No	Sydney, NSW	25/11/2003	*S. prolificans*		
62	WM 06.446	93/M	Tissue (ear)	Solid tumor malignancy, diabetes mellitus	I	No	Sydney, NSW	10/03/2003	*S. aurantiacum*		
63	WM 06.447	3/F	Tissue (brain)	None	I	Yes	Sydney, NSW	01/09/2003	*S. prolificans*		
64	WM 06.448	60/F	Bronchial washings	Chronic renal disease	C	Yes	Sydney, NSW	02/07/2003	*S. prolificans*		
65	WM 06.449	10/F	Ear swab	None	C	No	Sydney, NSW	11/07/2003	*S. prolificans*		
66	WM 06.450	58/F	Bronchial wash fluid	Chronic lung disease	C	Yes	Sydney, NSW	12/05/2003	*S. apiospermum*		
67	WM 06.451	62/F	Nose swab	None	C	Yes	Sydney, NSW	08/12/2003	*S. prolificans*		

Patient	WM no.	Age/Sex	Specimen type	Comorbidity/risk factor	Status	Building renovation	City/State	Date of isolation†	Species	Comments	Strain profile
68	WM 06.452	60/F	Sputum	None	C	Yes	Sydney, NSW	04/11/2003	*S. prolificans*		
69	WM 06.453	13/F	BAL	NA	C	No	Sydney, NSW	01/09/2003	*S. prolificans*		
70	WM 06.454	11/F	Sputum	NA	C	No	Sydney, NSW	01/09/2003	*S. aurantiacum*		
71	WM 06.455	57/M	Bronchial wash fluid	Lung transplantation	C	No	Sydney, NSW	19/12/2003	*S. apiospermum*		
72	WM 06.456	NA	Ear swab	NA	C	No	Sydney, NSW	08/01/2004	*S. apiospermum*		
73	WM 06.457	59/F	Blood	Hematological malignancy	I	Yes	Sydney, NSW	24/01/2004	*S. prolificans*	Suspected Sydney outbreak	
	WM 06.458	59/F	Sputum	Hematology malignancy	I	Yes	Sydney, NSW	24/01/2004	*S. prolificans*	Suspected Sydney outbreak	
74	WM 06.459	80/F	Ear swab	None	C	No	Sydney, NSW	28/01/2004	*S. aurantiacum*		
75	WM 04.468	61/M	Bronchial wash fluid	Solid tumor malignancy	C	No	Melbourne, VIC	12/01/2004	*S. prolificans*		
76	WM 04.497	71/F	Tissue (sphenoid sinus)	Chronic sinusitis	C	No	Sydney, NSW	24/02/2004	*S. aurantiacum*		
77	WM 06.462	30/F	Sputum	Cystic fibrosis	C	No	Sydney, NSW	16/02/2004	*S. aurantiacum*		
78	WM 06.463	55/F	Bronchial wash fluid	Recent surgery	C	No	Sydney, NSW	04/03/2004	*S. prolificans*		
79	WM 06.464	63/M	Tissue (eye)	Recent eye surgery	I	Yes	Sydney, NSW	01/03/2004	*S .prolificans*		
80	WM 06.465	29/F	Sputum	Diabetes mellitus	I	No	Sydney, NSW	15/04/2004	*S. aurantiacum*		Strain 80a
	WM 06.466	29/F	Tissue (bone)	Diabetes mellitus	I	No	Sydney, NSW	07/07/2004	*S. aurantiacum*		Strain 80a
	WM 06.467	29/F	Tissue (bone)	Diabetes mellitus	I	No	Sydney, NSW	12/08/2004	*S. aurantiacum*		Strain 80a
	WM 06.468	29/F	Wound fluid	Diabetes mellitus	I	No	Sydney, NSW	19/10/2004	*S. aurantiacum*		Strain 80b
81	WM 06.469	66/M	Ear swab	Diabetes mellitus	I	No	Sydney, NSW	19/04/2004	*S. apiospermum*		
82	WM 06.470	37/F	Sputum	Other immuno-compromise	C	No	Sydney, NSW	02/03/2004	*S.prolificans*		
83	WM 06.471	43/M	BAL	Lung transplantation	I	No	Sydney, NSW	15/05/2004	*S. apiospermum*		Strain 83a
	WM 06.472	43/M	Bronchial wash fluid	Lung transplantation	I	No	Sydney, NSW	13/05/2004	*S. apiospermum*		Strain 83a
	WM 06.473	43/M	BAL	Lung transplantation	I	No	Sydney, NSW	04/08/2004	*S. prolificans*		
	WM 06.474	43/M	BAL	Lung transplantation	I	No	Sydney, NSW	04/08/2004	*S. apiospermum*		Strain 83b
	WM 06.475	43/M	BAL	Lung transplantation	I	No	Sydney, NSW	05/08/2004	*S. apiospermum*		Strain 83b

Patient	WM no.	Age/Sex	Specimen type	Comorbidity/risk factor	Status	Building renovation	City/State	Date of isolation†	Species	Comments	Strain profile
84	WM 06.476	18/M	Sputum	Chronic lung disease	C	No	Sydney, NSW	08/03/2004	*S. aurantiacum*		
85	WM 06.477	NA	Ear swab	None	C	No	Sydney, NSW	09/05/2004	*S. apiospermum*		
86	WM 06.478	NA	Blood	NA	I	No	Sydney, NSW	03/06/2004	*S. prolificans*		
87	WM 06.479	18/F	Sputum	Cystic fibrosis	C	No	Sydney, NSW	09/07/2004	*S. aurantiacum*		
	WM 06.480	18/F	Sputum	Cystic fibrosis	C	No	Sydney, NSW	18/10/2004	*S. aurantiacum*		
	WM 06.481	18/F	Sputum	Cystic fibrosis	C	No	Sydney, NSW	18/11/2004	*S. aurantiacum*		
88	WM 06.482	24/M	Lung	None	I	No	Sydney, NSW	01/06/2004	*S. aurantiacum*		
89	WM 06.483	NA	Skin	None	C	No	Sydney, NSW	04/08/2004	*S. aurantiacum*		
90	WM 06.484	36/F	Sputum	Lung transplantation	C	No	Sydney, NSW	03/08/2004	*S. aurantiacum*		
91	WM 06.485	67/M	Sputum	Chronic lung disease	C	No	Sydney, NSW	23/08/2004	*S. prolificans*		
	WM 06.486	67/M	Sputum	Chronic lung disease	C	No	Sydney, NSW	23/08/2004	*S. apiospermum*		
92	WM 06.488	42/F	skin	None	C	No	Sydney, NSW	07/06/2004	*S. apiospermum*		
93	WM 06.489	43/M	Ear swab	None	C	No	Sydney, NSW	07/06/2004	*S. apiospermum*		
94	WM 06.490	53/F	Ear swab	Diabetes mellitus	C	No	Sydney, NSW	04/06/2004	*S. apiospermum*		
95	WM 06.491	71/M	Ear swab	None	C	No	Sydney, NSW	04/06/2004	*S. apiospermum*		
96	WM 06.492	70/F	Sputum	Chronic lung disease	C	No	Sydney, NSW	17/06/2004	*S. aurantiacum*		
97	WM 06.493	5/M	Ear swab	None	C	No	Sydney, NSW	17/06/2004	*S. aurantiacum*		
98	WM 06.494	26/F	Ear swab	None	C	No	Sydney, NSW	10/06/2004	*S. apiospermum*		
99	WM 06.495	76/F	Tissue (maxillary sinus	None	C	No	Sydney, NSW	17/06/2004	*S. aurantiacum*		Strain 99a
	WM 06.496	76/F	Tissue (maxillary sinus	None	C	No	Sydney, NSW	17/06/2004	*S. aurantiacum*		Strain 99b
100	WM 06.498	59/F	Ear swab	None	C	No	Sydney, NSW	20/07/2004	*S. aurantiacum*		
101	WM 06.499	15/M	Sputum	Cystic fibrosis	C	No	Sydney, NSW	15/07/2004	*S. prolificans*		
102	WM 06.500	62/F	Sputum	Chronic lung disease	C	No	Sydney, NSW	22/072004	*S. apiospermum*		
	WM 06.501	62/F	Sputum	Chronic lung disease	C	No	Sydney, NSW	22/07/2004	*S. prolificans*		
103	WM 06.502	34/M	Tissue (ethmoid sinus)	Chronic sinusitis	I	No	Sydney, NSW	16/08/2004	*S. prolificans*		
	WM 06.503	34/M	Tissue (ethmoid sinus)	Chronic sinusitis	I	No	Sydney, NSW	31/08/2004	*S. prolificans*		

Patient	WM no.	Age/Sex	Specimen type	Comorbidity/risk factor	Status	Building renovation	City/State	Date of isolation†	Species	Comments	Strain profile
104	WM 06.504	55/F	Ear swab	None	C	No	Sydney, NSW	27/08/2004	*S. apiospermum*	White colony	
	WM 06.505	55/F	Ear swab	None	C	No	Sydney, NSW	27/08/2004	*S. apiospermum*	Grey colony	
105	WM 06.506	56/M	Nasal swab	None	C	No	Sydney, NSW	03/09/2004	*S.prolificans*		
106	WM 06.507	30/M	Ear swab	None	C	No	Sydney, NSW	03/09/2004	*S. prolificans*		
107	WM 06.508	8/M	Ear swab	None	C	No	Sydney, NSW	01/09/2004	*S. prolificans*		
108	WM 06.509	28/M	Ear swab	None	C	No	Sydney, NSW	25/08/2004	*S. apiospermum*		
109	WM 06.511	82/F	Sputum	None	C	No	Sydney, NSW	13/08/2004	*S. aurantiacum*		
110	WM 06.512	7/M	Ear swab	None	I	No	Sydney, NSW	06/09/2004	*S. prolificans*		
111	WM 06.513	45/M	BAL	Lung transplantation	I	No	Melbourne, VIC	19/12/2003	*S. prolificans*		
112	WM 06.514	47/M	BAL	Lung transplantation	C	No	Melbourne, VIC	28/01/2004	*S. prolificans*		
113	WM 06.515	44/M	Blood	Hematological malignancy	I	No	Melbourne	09/03/2004	*S. prolificans*		
114	WM 06.516	61/M	BAL	Lung transplantation	C	No	Melbourne, VIC	27/04/2004	*S. prolificans*		
115	WM 06.517	53/F	Sputum	Cystic fibrosis	C	No	Melbourne, VIC	08/09/2004	*S. prolificans*		
116	WM 06.518	64/F	Sputum	Lung transplantation	C	No	Melbourne, VIC	12/11/2003	*S. prolificans*		
117	WM 06.519	66/M	BAL	Lung transplantation	C	No	Melbourne, VIC	29/09/2004	*S. prolificans*		
118	WM 06.520	62/M	Tissue (chest)	Lung transplantation	I	No	Sydney, NSW	15/10/2004	*S. apiospermum*		Strain 118a
	WM 06.521	62/M	Pleural fluid	Lung transplantation	I	No	Sydney, NSW	14/10/2004	*S. apiospermum*		Strain 118a
	WM 06.522	62/M	Tissue (bone)	Lung transplantation	I	No	Sydney, NSW	17/10/2004	*S. apiospermum*		Strain 118a
	WM 06.523	62/M	Wound fluid	Lung transplantation	I	No	Sydney, NSW	02/11/2004	*S. apiospermum*		Strain 118b
	WM 06.524	62/M	Tissue (bone)	Lung transplantation	I	No	Sydney, NSW	09/11/2004	*S. apiospermum*		Strain 118b
119	WM 06.525	35/M	Blood	Hematological malignancy	I	No	Perth, WA	01/12/2003	*S. prolificans*		
	WM 06.526	35/M	Skin	Hematological malignancy	I	No	Perth, WA	27/11/2003	*S. prolificans*		

*C, colonization; QLD, Queensland; BAL, bronchoalveolar lavage; SA, South Australia; I, infection; ACT, Australian Capital Territory; VIC, Victoria; NSW, New South Wales; HSCT, hematopoeitic stem cell transplant; WA, Western Australia; NA, not available.

†Isolation dates are given where known; otherwise, only month, year, or both is stated.
