# Supplementary figures and images for: Molecular Typing of Australian Scedosporium Isolates Showing Genetic Variability and Numerous S. aurantiacum
Source: Emerg Infect Dis. 2008 Feb;14(2):282–90. doi: 10.3201/eid1402.070920 (PMC2600218; doi:10.3201/eid1402.070920)

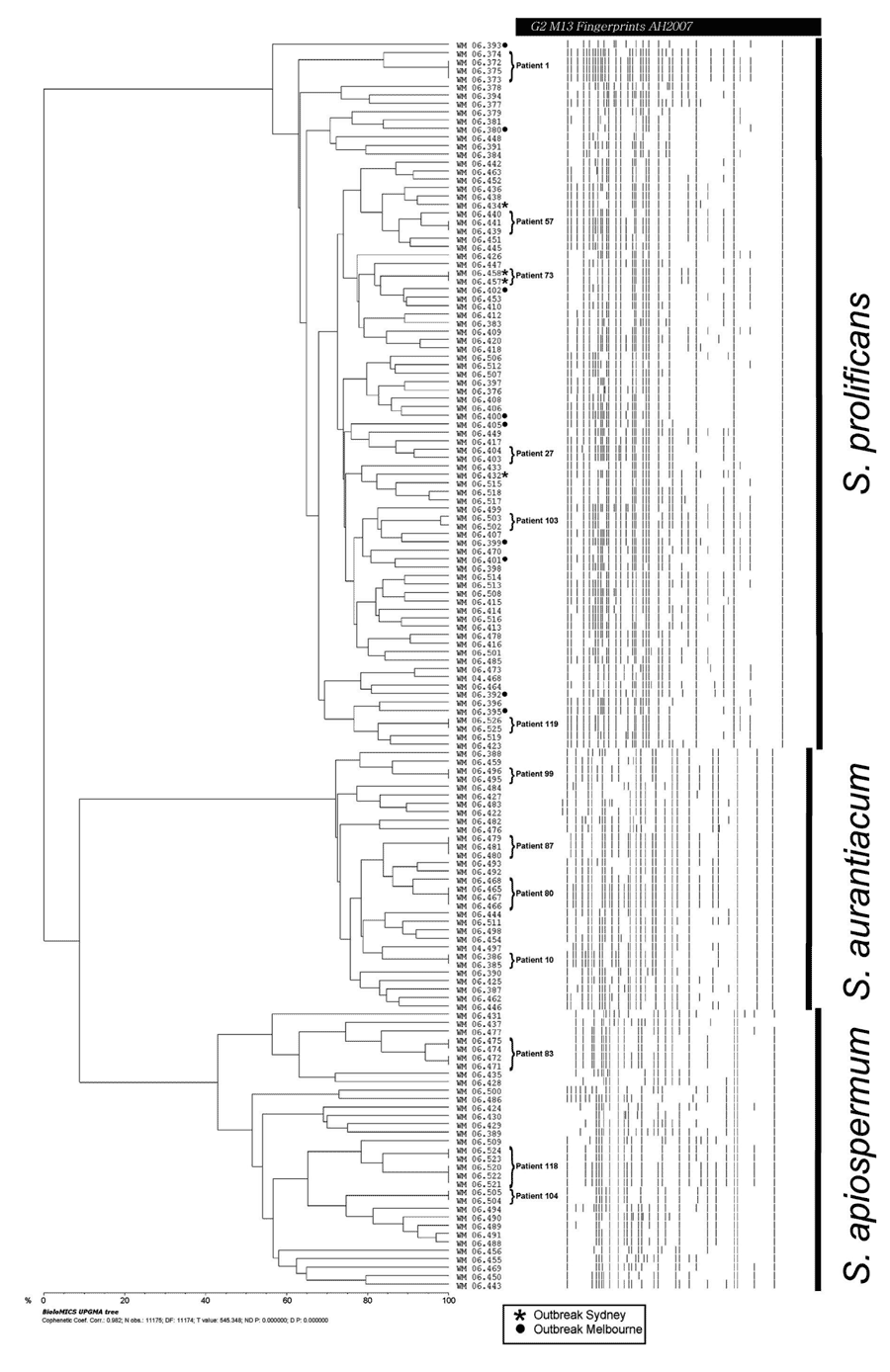

Supplement: Appendix Figure 1 — Dendogram generated from the PCR fingerprinting profiles obtained with the microsatellite primer M13 for all investigated Scedosporium isolates. The dendogram was designed by using the unweighted pair group method with arithmetic mean and the procedure of Nei and Li (32) in the program BioloMICS version 7.5.30. Pt, patient. [file 07-0920_app1-s2.gif]

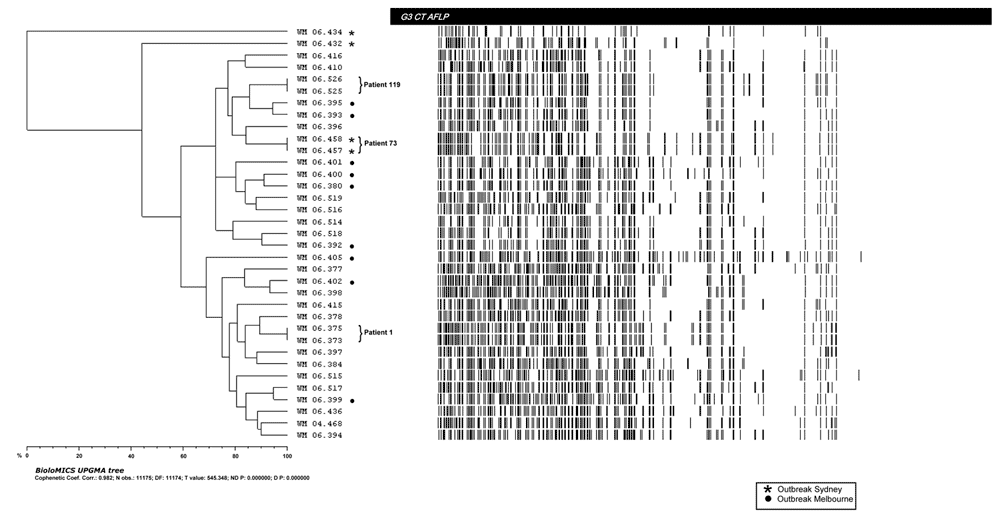

Supplement: Appendix Figure 2 — Dendogram generated by unweighted pair group method with arithmetic mean and the procedure of Nei and Li (32) for the amplified fragment length polymorphism (AFLP) profile obtained from the 2 suspected case clusters and selected 23 other Australian Spedosporium prolificans strains using the primer pairs EcoRI-TG and MseI-CA. None of the investigated isolates showed any epidemiologic connection except the isolates obtained from the same patient (nos. 1, 73, 119). Pt, patient. [file 07-0920_app2-s3.gif]
